# Effect of caregiver burden on anticipatory grief among caregivers of elderly cancer patients: Chain mediation role of family functioning and resilience

**DOI:** 10.3389/fpsyg.2022.1020517

**Published:** 2023-01-10

**Authors:** Caiyue Li, Nan Tang, Lili Yang, Qing Zeng, Tana Yu, Xiaojin Pu, Juan Wang, Hongchen Zhang

**Affiliations:** ^1^School of Nursing, Lanzhou University, Lanzhou, China; ^2^Lanzhou University First Hospital, Lanzhou, China; ^3^Lanzhou University Second Hospital, Lanzhou, China

**Keywords:** cancer, caregivers, anticipatory grief, caregiver burden, family functioning, resilience

## Abstract

This study aimed to explore the relationship between caregiver burden and anticipatory grief among caregivers of elderly cancer patients, and to examine the chain mediation effects of family functioning and resilience. A total of 624 valid questionnaires were collected. The Structural Equation Model was established to test the mediating effects of family functioning and resilience. Results showed that caregiver burden has a direct positive effect on anticipatory grief, both family functioning and resilience have negative effects on caregiver burden and anticipatory grief, and that resilience moderates the mediating effect of family functioning. Our findings suggest that reducing the caregiver burden among caregivers of elderly cancer patients, improving family functioning, and enhancing resilience have important effects in alleviating the anticipatory grief of caregivers. Our findings provide some references for further research. Medical staff should better understand the grief experience of caregivers and implement interventions to enable caregivers to better cope with anticipatory grief and psychological stress, so as to promote the quality of care for elderly cancer patients.

## Introduction

Cancer is one of the major diseases threatening human health, and its incidence has shown a progressive increase over the years (Sung et al., 2021). Caregivers of cancer patients, from the time of diagnosis to the deterioration of the disease, experience a series of physiological and psychological stress reactions, including the anticipation of death resulting in anticipatory grief (Yu et al., 2021). Anticipatory grief is commonly defined as the mourning, coping, planning, and psychological reorganization of one’s life in response to an impending loss as well as the past, present, and future losses (Rando, 1986). This phenomenon acts as a “safe-guard” against the impact of sudden death of patients with life-threatening diseases on family caregivers, who are likely to express a mixture of calm and relief after the death due to this emotional preparation (Sweeting and Gilhooly, 1990). However, it can also be a contributor to the psychological distress among caregivers (Seyedfatemi et al., 2021). Anticipatory grief can lead to physiological, psychological, cognitive, and mental problems for caregivers, including insomnia, headache, anxiety, anger, forgetfulness, depression (Liew et al., 2019), and other symptoms, and even suicidal tendencies in severe cases (Coelho et al., 2018). Thus, it is especially important for caregivers to appropriately cope with the impending death of a loved one. At the same time, timely assessment of the psychological state of caregivers by medical staff can enable caregivers to improve their ability to cope with care burden.

Caregiver burden is described as a biopsychosocial strain experienced by the caregiver due to caring for a family member or a loved one over time (Liu et al., 2020), which includes the personal strain and role strain. Personal strain refers to a person’s self-perception of caregiving difficulties. Caregivers of elderly cancer patients typically devote a lot of energy and time to caring for patients while rarely caring for themselves, which can lead to a decline in their physiological and psychological health (Song et al., 2011). Role strain refers to the negative reaction to the caregivers’ social, occupational, and family roles. Chronic exposure to role strain may result in poor well-being, social isolation, and financial challenges (Campbell et al., 2014). Additionally, previous studies have identified other adverse effects of caregiver burden, such as reduced quality of care provided (Bastawrous, 2013), and increased intensity of anticipatory grief (Axelsson et al., 2020).

Family functioning is described as how well family members communicate, fulfill family responsibilities, accept family routines, and cope with and adjust to family stress (Zhang, 2018). Five types of families (supportive, conflict-resolving, intermediate, sullen, and hostile family) illustrate the different degrees of family functioning. Individuals in a supportive family or conflict-resolving family have greater ability to respond to challenges, are more pleased with their emotional connections, and perceive a higher quality of life, all of which contribute to active coping with a family crisis (Huang et al., 2022). Family functioning is also a protective factor during the grief process (Smilkstein, 1978). Good family functioning can help caregivers wane over the grief in 13 months (Kissane et al., 1998).

Resilience has various definitions in extant theoretical writings. Rutter (2012) emphasized that resilience was a dynamic process encompassing positive adaption in response to major adversity. Connor and Davidson (2003) defined resilience as a personal characteristic that enables people to resume normal functioning in the face of significant adversity and traumatic psychosocial events. In this study, resilience was seen as a personal trait, which has a positive effect on physiological, psychological (Dionne-Odom et al., 2021), and social function (Luo et al., 2020). Exposure of individuals to chronic psychological stressors can lead to deterioration of their health and accelerate aging (Canevelli and Bersani., 2022). People with high resilience could perceive themselves to cope with stress more resourceful and less overwhelmed by stressors (García-León et al., 2019). Additionally, strengthening resilience of caregivers and its associated psychological variables (e.g., optimism) was shown to reduce the stress caused by biological, psychological, social, and spiritual changes, and improve caregivers’ role adaptation (Palacio et al., 2020).

Studies have shown that caregiver burden can cause family problems and family conflicts (Jeong et al., 2020), and may negatively affect resilience (Li et al., 2018). Anticipatory grief may be reduced by increasing family functioning (Chiu et al., 2010), and by increasing resilience (Vegsund et al., 2019). Because talking about death is a taboo in China, caregivers seldom communicate illness and death with patients, and always make decisions based on their understanding of the disease and social customs. The resultant poor communication between caregivers and patients tends to increase caregiver burden as well as their anticipatory grief (Yu et al., 2021). However, this ignores the internal mediating effects of caregiver burden and anticipatory grief. Furthermore, no study has concomitantly explored the role of both family functioning and resilience in mediating the effects of caregiver burden on anticipatory grief.

Therefore, this study aimed to investigate the role of family functioning and resilience to better characterize the mechanism by which caregiver burden is translated to anticipatory grief. We conducted the study in a sample of caregivers of elderly cancer patients. Firstly, cancer is a leading cause of morbidity and mortality in older population. Although death is expected, it is often experienced as too sudden, and there is a high incidence of anticipatory grief among caregivers of elderly cancer patients (Coelho et al., 2018). Secondly, caregivers of elderly cancer patients are often also older persons, and approximately a third of them are in a bad health condition (Balducci, 2019). Caregivers of elderly cancer patients play an important role in improving the outcome of cancer treatment and quality of life, which may cause a huge caregiver burden (Deshields et al., 2012). According to a systemic review, the prevalence of caregiver burden among caregivers of elderly cancer patients ranges from 1% to more than 35% (Dionne-Odom et al., 2017). Thirdly, caregivers of elderly cancer patients represent a vulnerable caregiver group, but there are few evidence-based intervention resources available to support the demands and challenges of cancer caregiving (Sun et al., 2019). Identifying the relationship between caregiver burden, family functioning, resilience, and anticipatory grief in caregivers of elderly cancer patients may help inform interventions to improve their coping ability for anticipatory grief.

## Theoretical foundations and hypotheses development

### Conservation of resources theory

Despite considerable evidence on the downsides of caregiver burden, the mechanism through which caregiver burden exerts its effects on anticipatory grief is still under exploration. Various theoretical models that elucidate potential mechanisms of caregiver burden have been proposed, including but not limited to experiencing some sort of stress and overload (Buglass, 2010); multiple losses (Holley and Mast, 2009); and depletion of coping resources (Boerner and Schulz, 2009). Given caregiver burden and anticipatory grief have potential adverse outcomes among caregivers of elderly cancer patients, more in-depth research on the relationship between them is warranted to inform the precise interventions to improve caregivers’ negative emotions. Our study is based on the framework of the conservation of resources (COR) theory. It is essentially a theory of motivation that provides a framework for understanding the associations among the consequences of major and traumatic stress. According to this theory, people are inherently motivated to obtain, protect, and pursue the acquisition of resources. Actual loss or threat of loss of resources and lack of accrual of adequate benefits from resource investments result in stress (Snyder et al., 2020). This suggests that individuals perceive loss of resources as an external threat, and they do their best to acquire and maintain available resources, thus making self-adjustment to the environment, and promoting their mental health.

According to the COR theory, the resources include object resources, conditions, personal characteristics, and energy. In addition, social relationship is an important part of accessing these resources (Hobfoll Stevan, 1989). Object resources include assets, salaries, and similar items. Condition resources include marriage, seniority, and employment. Personal characteristics include positive personality traits and psychological resources such as resilience, self-esteem. Energy resources include time, knowledge, and learning ability. Personal characteristics and social relationship are regarded as the resources that facilitate the preservation of valuable resources. Inspired by this theory, this study considers caregiver burden as a stressful event for caregivers, family functioning as a representative variable of social relationship, resilience as a representative variable of personal characteristics, and anticipatory grief as an emotional response to the loss. The purpose of the study was to explore the effect of family functioning and resilience in mediating the relationship between caregiver burden and anticipatory grief of caregivers of elderly cancer patients. Application of this theory in a variety of circumstances has shown that people with adequate resources tend to cope better with stress (Sumer et al., 2005; Merino et al., 2021).

### Caregiver burden and anticipatory grief

Caregiver burden is essentially related to anticipatory grief. Caregiver burden affects anticipatory grief due to the resultant physiological and psychosocial stress, which has a detrimental effect on health, social life, and economic status (Yu et al., 2021). Drown into a caregiving role, caregivers require unremitting dedication to demands of the patients. The burden of caring has been identified as an important cause of damage to caregivers’ physical and mental health, and it may subsequently lead to loss and grief experiences (Al-Gamal and Long, 2014). In addition, taking up a caregiver role for a patient diagnosed with cancer, caregivers may experience more changes in their life and suffer from higher mental tension, for example, denial, anxiety, depression, uncertainty, and fear, which limits their ability to participate in social and recreational activities and to remain highly involved in their own occupational and family roles, causing them to feel lost the primary control over life (Noyes et al., 2010). These emotional reactions are considered as the main psychological expressions of anticipatory grief (Al-Gamal and Long, 2010). Having such losses due to being a caregiver has been found a positive association with anticipatory grief in previous studies (Holley and Mast, 2009; Cheng et al., 2019). Based on the above empirical studies, we proposed the following hypothesis:

*H1*: Caregiver burden is positively associated with their anticipatory grief.

### Family functioning as mediator between caregiver burden and anticipatory grief

Caregiver burden may result in impaired family functioning. Caregivers perceive an imbalance in the distribution of caregiving responsibilities due to family members regarding the caregiving provision as the specific responsibility of a particular individual (Jeong et al., 2020). In addition, dissatisfaction with the amount and quality of attention accorded to a patient may cause family conflicts (Pearlin et al., 1990). Besides, the caregiver burden also causes relationship deprivation between the caregiver and patients. The relational deprivation is also a manifestation of poor family functioning, and leads to different levels of anticipatory grief (Noyes et al., 2010). With worsening of the patients’ illness, caregivers and patients are unable to interact normally and maintain the previous communication style. Moreover, caregivers tend to spend less time together in family activities, which prevents the establishment of a close, intimate relationship among them.

Family functioning as a mediating role can weaken the effect of caregiver burden on anticipatory grief. A well-functioning family is characterized by open communication among family members and freedom to express feelings, which facilitates adaptive adjustment and grief resolution among the members (Delalibera et al., 2015). A previous study demonstrated the important role of family functioning in relieving anticipatory grief (Li et al., 2019). Family members can provide practical support (such as financial support) and emotional support (such as encouragement and hope) by working together to manage demands (Zhao et al., 2021), this can help reduce the level of physiological and mental exhaustion among caregivers, and reduce the risk of depression and anxiety (Shin et al., 2018). Therefore, caregivers with good family functioning can reduce the grieving process by receiving beneficial emotional responses and available assistance. Based on the discussion above, we proposed the following hypotheses:

*H2*: Caregiver burden is negatively associated with their family functioning.

*H3*: Family functioning moderates the positive relationship between caregiver burden and anticipatory grief, in that the higher the family functioning, the weaker is the positive relationship between caregiver burden and anticipatory grief.

### Resilience as mediator between caregiver burden and anticipatory grief

There is a general consensus that caregiver burden is negatively related to resilience (Hoang et al., 2018). Resilience as a protective element refers to a caregiver’s ability to adapt to the physiological and psychological demands of their role (McKenna et al., 2022). However, some caregivers adopt an avoidance style to deal with caregiver burden when experiencing challenges such as the high demands of caregiving (Junkins et al., 2020). At the same time, lack of adequate resources to cope with these challenges can inculcate negative emotions such as anxiety and depression, and may even disrupt their relationship with the patients (Noyes et al., 2010).

Resilience was found to negatively related to grief symptoms. This emphasizes the role of positive psychology in empowering human strength to cope with adversity. Individuals with high resilience can adjust successfully in the face of stressful events, including the loss of a loved one (Arizmendi and O’Connor, 2015). A systematic review found that resilience is associated with alleviated caregiver burden in end-of-life and palliative care (Palacio et al., 2020). However, it was unknown how resilience was associated with anticipatory grief symptoms. In general, the level of resilience was crucial for a healthy adaptation to grief. Therefore, the following hypotheses are proposed:

*H4*: Caregiver burden is negatively associated with their resilience.

*H5*: Resilience moderates the positive relationship between caregiver burden and anticipatory grief, in that the higher the resilience, the weaker is the positive relationship between caregiver burden and anticipatory grief.

### Family functioning to resilience

Previous studies have shown that a positive experience of family functioning can foster resilience (Kukihara et al., 2020). Family functioning refers to the feeling of being socially connected and a sense of belongingness among family members. Family functioning can foster love, trust, and encouragement for caregivers to increase their resilience (Hawkley et al., 2021). Therefore, the following hypothesis is proposed:

*H6*: Family functioning is positively associated with the resilience of caregivers.

## Materials and methods

### Research framework

**Figure 1:**
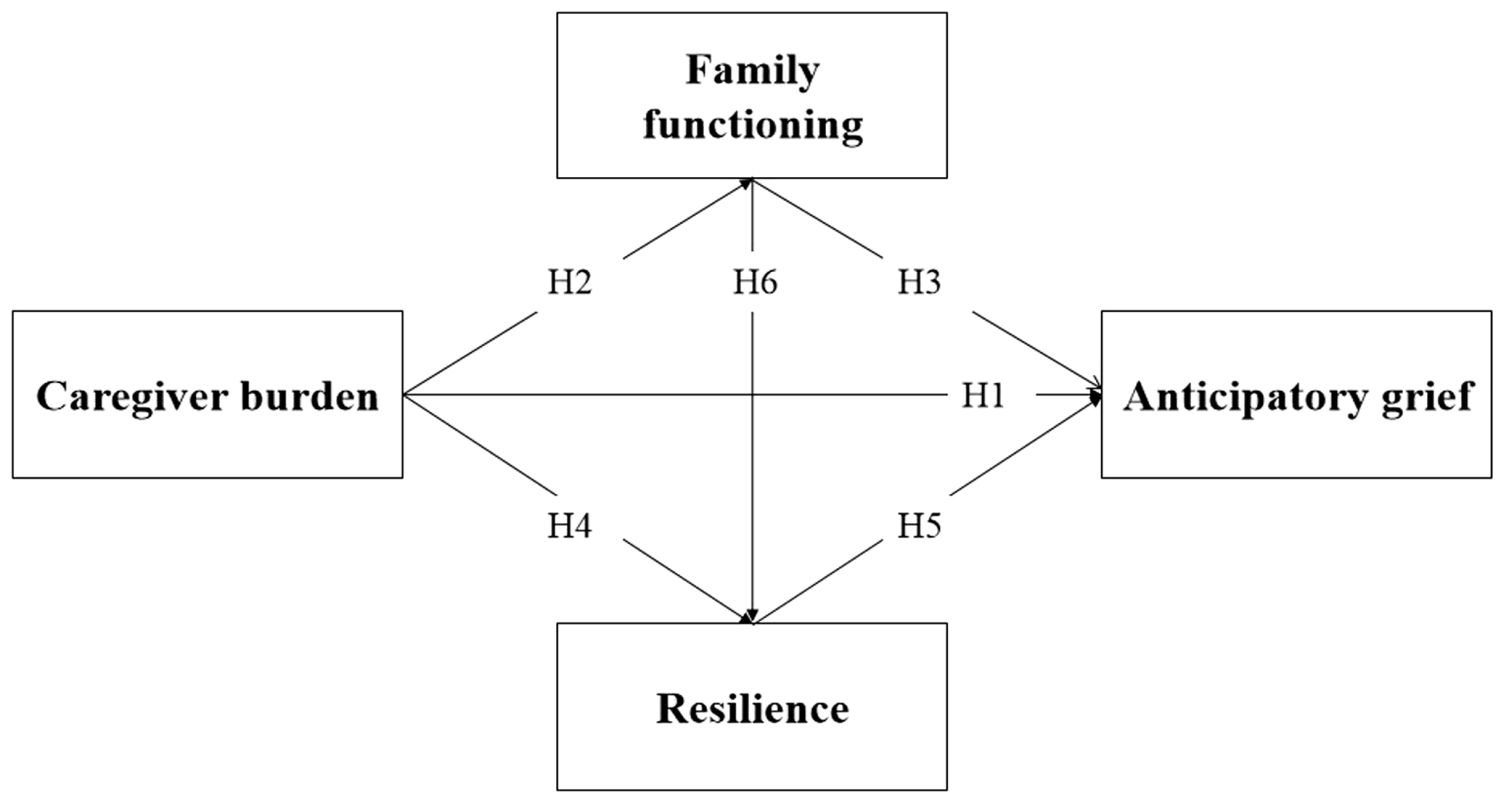
A hypothetical model of the relationship between variables. Influence of caregiver burden on anticipatory grief through family functioning and resilience.

It illustrates a hypothetical model of the relationship between the following variables, caregiver burden as an independent variable, anticipatory grief as a dependent variable, and family functioning and resilience as mediating variables. Based on the study hypotheses, the proposed research framework is presented in Figure 1.

### Participants and procedure

It was a descriptive cross-sectional research design, data were collected through a questionnaire survey conducted using a convenience sample of primary caregivers of elderly cancer patients. Patients diagnosed with cancer and aged ≥ 60 were recruited in this study. Caregiver’s inclusion criteria were as follows: (a) act as a primary caregiver (spouse, child, daughter-in-law, son-in-law, etc.); (b) be aware of the patient’s illness condition; and (c) volunteer for this study. We excluded caregivers who were unable to complete the questionnaire because of cognitive impairment, communication disorders, or weakness.

Researchers conducted unified training for three investigators from different hospitals who were required to contact participants and assist those who had difficulty understanding the questionnaires. After explaining the purpose of the study and obtaining informed consent from tumor ward managers and participants, investigators distributed the questionnaires to caregivers in a one-to-one manner. Questionnaires consisted of the scales of the Chinese version of Zarit Burden Interview, the family APGAR Questionnaire, the Connor-Davidson Resilience Scale, and the Anticipatory Grief Scale. Before the formal investigation, a preliminary investigation was conducted in a Class III Grade A hospital in Lanzhou city, China. The formal survey was carried out at three Class III Grade A hospitals in Lanzhou city, China between October 2019 and September 2020. The questionnaires were returned on the spot after completion. The total questionnaires took about 15 min to complete, and all participants were given small gifts after filling out the questionnaires as compensation. A total of 650 questionnaires were distributed and collected. After excluding incomplete questionnaires and outliers, the final valid sample size was 624 (valid response rate: 96%). The demographic characteristics of the study population are summarized in Table 1.

**Table 1:** Demographic characteristics of participants (*N* = 624).

Characteristics	Items	Frequency	Percentage (%)
Age	48.3 ± 11.4 years		
Gender	Male	331	53
	Female	293	47
Relationship with patients	Spouse	112	18
	Children	474	76
	Others	38	6
Education level	Primary school and below	75	12
	Secondary school	218	35
	High school	150	24
	College and above	181	29
Residence	City	349	56
	Countryside	275	44
Working status	Employed	205	33
	Unemployed	324	52
	Retired	94	15
Monthly income	≤ 3,000 Yuan	262	42
	3,001–6,000 Yuan	281	45
	≥ 6,001 yuan	81	13

## Measures

### Caregiver burden

Caregiver burden was assessed using the Zarit Burden Interview developed by Zarit et al. (1985). The scale consists of 22 items, including two subscales: personal strain and role strain. All items were scored on a five-point Likert scale ranging from 0 (mild caregiver burden) to 88 (strong caregiver burden). Higher scores reflect a higher level of caregiver burden. The present study adopted the Chinese version that is more suitable for Chinese caregivers of elderly cancer patients (Wang et al., 2006). The Cronbach’s α coefficient of this sample was 0.870, while those of two subscales were 0.890 and 0.830.

### Family functioning

Family functioning was assessed using the family APGAR Questionnaire developed by Smilkstein (1978), which has been translated and validated in China (Lv et al., 1999). The scale includes 5 items: adaption, partnership, growth, affection, and resolve. Each item was rated on a scale ranging from 0 (hardly ever experience) to 2 (often experience). All items are scored on a four-point Likert scale ranging from 0 to 10. The levels of family functioning are defined based on the total scores as follows: 0–3 indicates a low level of family functioning, 4–7 indicates a moderate level of family functioning, and 8–10 indicates a high level of family functioning. The questionnaire was shown to be reliable and has good test–retest validity (correlation coefficient: 0.800–0.830). The Cronbach’s α coefficient of the scale in the present sample was 0.910.

### Resilience

Resilience was assessed using the Connor-Davidson Resilience Scale (Connor and Davidson, 2003), which was translated into Chinese, and its psychometric properties were verified (Yu et al., 2007). It consists of 25 items, and 3 subscales: optimism, self-improvement, and toughness. All items are scored on a four-point Likert scale ranging from 0 to 100. A higher score on a certain demission indicates higher resilience. In this study, the Cronbach’s α coefficient of the overall scale was 0.910, and the Cronbach’s *α* coefficients of the subscales ranged from 0.730 to 0.890.

### Anticipatory grief

Anticipatory grief was assessed using the Chinese version of the Anticipatory Grief Scale, which was developed by Theut et al. (1991) and modified by Zhou et al. (2017). It consists of 27 items and 7 subscales: guilty, anger, anxiety, irritability, sadness, feeling of loss, and decreased ability to function at usual tasks. All items are scored on a five-point Likert scale ranging from 27 (low anticipatory grief) to 135 (high anticipatory grief). Higher scores reflected a higher level of anticipatory grief. The Cronbach’s α coefficient of this sample was 0.870 and those of three subscales ranged from 0.840 to 0.910.

### Data analysis

Means, standard deviations, and correlations of the variables were computed using SPSS 24.0. Multiple regression analysis was used to analyze the associations between the caregiver burden and anticipatory grief. We controlled caregiver’s age, gender, and relationship with patients, and then conducted with two types of caregiver burden as independent variables and anticipatory grief and its seven different dimensions as dependent variables. Besides, the role of family functioning and resilience as mediators was tested *via* Structural Equation Modeling (SEM) using AMOS 23.0, which is a multivariate technique to analyze the observed and latent variables relationships. It was similar to a mixture of both factor analysis and multivariate regression analysis.

We applied the two-step procedure of SEM using AMOS 23.0. Firstly, we calculated the measurement model and obtained results. Secondly, we assessed the overall fit of the model of data to examine the structural model. The goodness-of-fit indices were used to test the hypothesis model, and generally *χ*^2^/degrees of freedom (*df*) < 5, root mean square error of approximation (RMSEA) < 0.08, comparative fit index (CFI) > 0.9, Tucker-Lewis index (TLI) > 0.9, and Standardized root mean square residual (SRMR) < 0.05 indicated a good overall fitness of the structural model. The mediation effects of family functioning and resilience were tested by using bootstrapping procedures in AMOS 23.0.

## Results

### Descriptive statistics and analysis of the correlations between variables

Means, standard deviations, and correlations of all the study variables are shown in Table 2. There was a significant correlation between caregiver burden, family functioning, resilience, and anticipatory grief. Caregiver burden showed a positive association with anticipatory grief. Family functioning and resilience showed a negative association with anticipatory grief. In turn, family functioning and resilience showed a negative association with caregiver burden. Family functioning showed a positive association with resilience. The results of the above correlation analysis suggest that it is suitable for the subsequent mediating effect analysis, and it also lays a foundation for further hypothesis testing.

**Table 2:** Means, standard deviations (SD), and intercorrelations among study measures.

Measure	Mean	SD	1	2	3
1. Caregiver burden	38.32	11.05			
2. Family functioning	5.14	1.28	−0.49^**^		
3. Resilience	58.49	12.08	−0.43^**^	0.31^*^	
4. Anticipatory grief	88.49	10.65	0.52^**^	−0.51^**^	−0.38^**^

^**^Correlation is significant at the 0.01 level (2-tailed); ^*^Correlation is significant at the 0.05 level (2-tailed).

### Multiple regression analysis between caregiver burden and anticipatory grief

In the multivariate analysis, the results found that the variables “personal strain” and “role strain” increased, and the odds of feeling anticipatory grief were significantly increased when holding the other independent variables (e.g., age, gender, and relationship with patient). Both personal strain and role strain were found to be positively correlated with each item of anticipatory grief, and the caregiver burden was significant contributor to anticipatory grief (Table 3).

**Table 3:** Multiple regression analysis of each dimension of caregiver burden to anticipatory grief (age, relationship with patients were statistically controlled).

Dependent variable	Independent variable	*B*	SE	*t*	*P*
Anticipatory grief	Personal strain	0.648	0.094	6.682	< 0.001
	Role strain	1.440	1.038	3.547	< 0.001
Sadness	Personal strain	0.527	1.384	2.145	< 0.001
	Role strain	0.436	2.114	1.398	< 0.001
Feeling of loss	Personal strain	0.547	0.964	2.374	0.024
	Role strain	0.368	0.853	3.481	< 0.001
Anger	Personal strain	0.429	1.622	4.210	< 0.001
	Role strain	0.341	1.915	3.896	< 0.001
Irritability	Personal strain	0.513	2.061	4.518	0.020
	Role strain	0.378	1.931	2.947	< 0.001
Anxiety	Personal strain	0.354	0.861	3.077	0.014
	Role strain	0.419	0.766	2.581	0.006
Guilty	Personal strain	0.286	0.628	4.263	0.017
	Role strain	0.294	0.833	4.013	0.035
Decreased ability to function at usual tasks	Personal strain	0.632	0.950	2.950	< 0.001
	Role strain	0.548	0.764	3.811	0.031

*R*^2^ = 0.419; Adjusted *R*^2^ = 0.36; *F* = 6.343, *p* < 0.001.

### Testing the mediating effects of caregiver burden and anticipatory grief

**Figure 2:**
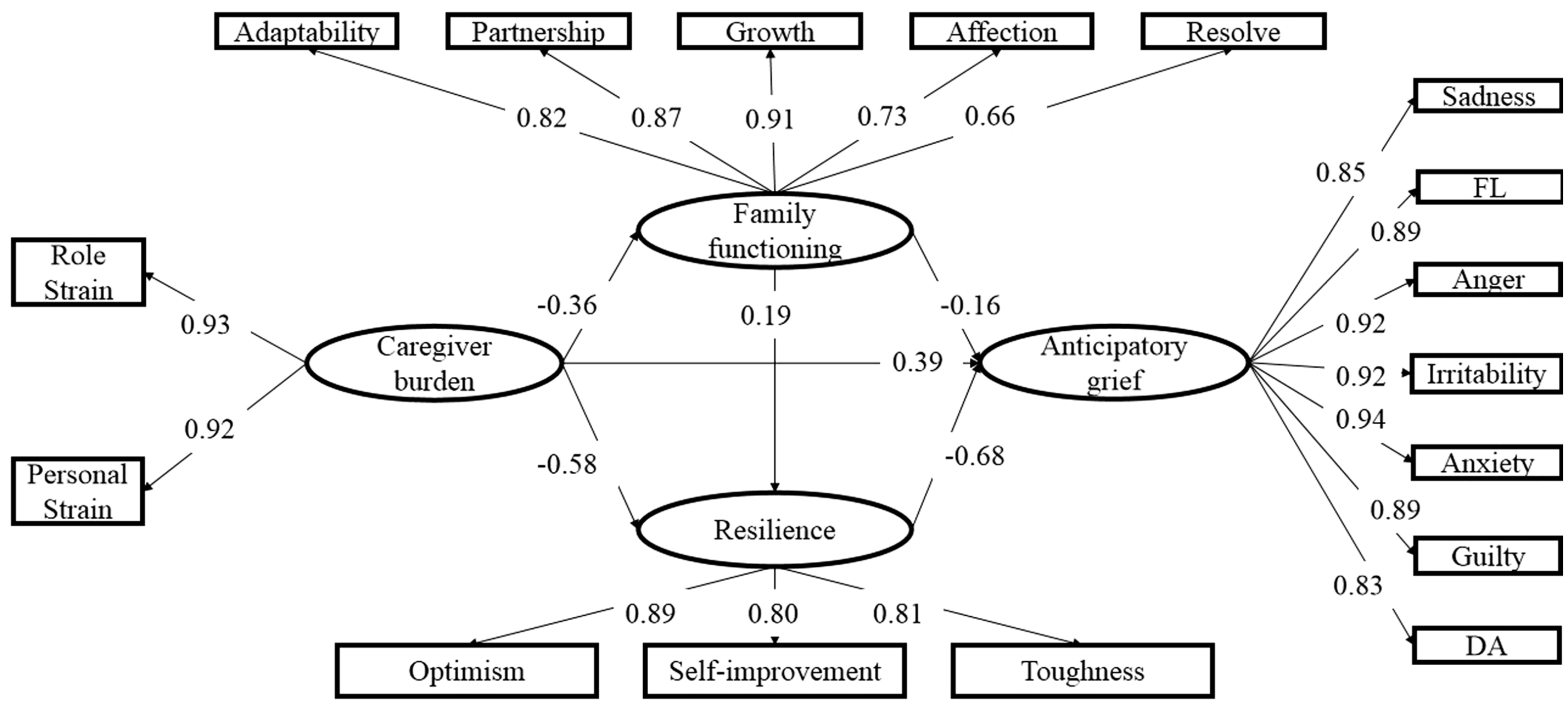
The Structural Equation Model linking caregiver burden and anticipatory grief through family functioning and resilience. Factors loadings are standardized. FL = feeling of loss, DA = decreased ability to function at usual tasks.

The hypothesized path model of this study comprised 17 observed variables and 4 latent variables (caregiver burden, family functioning, resilience, and anticipatory grief). This model showed an excellent fit with the data: *χ*^2^/degrees of freedom (*df*) = 2.37 < 5, *p* < 0.001, RMSEA = 0.05 < 0.08, CFI = 0.92 > 0.9, TLI = 0.95 > 0.9, and SRMR = 0.04 < 0.05. The results showed that the direct path coefficients in the proposed directions were significant, and indicated that family functioning and resilience partially mediated the relationship of caregiver burden to anticipatory grief (Figure 2).

Using the original data set (*N* = 624), we generated 2000 bootstrapping samples by random sampling. The mediating effects of family functioning and resilience, and their 95% confidence intervals (CI) are shown in Table 4. 95% CI values of indirect effects indicated that family functioning and resilience significantly mediated the relationship between caregiver burden and anticipatory grief. Thus, findings support that family functioning and resilience all have a partial mediating role in the effect of caregiver burden and anticipatory grief among caregivers of elderly cancer patients.

**Table 4:** Bootstrapping direct and indirect effects and 95% confidence intervals (CI) for the mediational model.

Model pathways	Estimated	95%CI	
		Lower	Upper
Caregiver burden → Anticipatory grief	0.39	0.30	0.50
Caregiver burden → Family functioning → Anticipatory grief	0.06	0.02	0.08
Caregiver burden → Resilience → Anticipatory grief	0.39	0.18	0.40
Caregiver burden → Family functioning → Resilience → Anticipatory grief	0.05	0.02	0.07

The direct path from caregiver burden to family functioning was significant (*β* = − 0.36, *p* < 0.05), as was the direct path from family functioning to anticipatory grief (*β* = − 0.16, *p* < 0.05). Moreover, the indirect path (path 2) from caregiver burden to anticipatory grief through family functioning was significant (effect = 0.06, 95%CI = [0.02,0.08]). Thus, we suggest that there was a mediating effect of family functioning existed between caregiver burden and anticipatory grief.

Additionally, the path from caregiver burden to resilience was significant (*β* = − 0.58, *p* < 0.05), as was the direct path from resilience to anticipatory grief (*β* = − 0.68, *p* < 0.05). Moreover, the indirect path (path 3) from caregiver burden to anticipatory grief through resilience was significant (effect = 0.39, 95%CI = [0.18,0.40]). Thus, we suggested that there was a mediating effect of resilience existed between caregiver burden and anticipatory grief.

## Discussion

Our study adopted a chain mediating model to explore the influence of caregiver burden, family functioning, and resilience on anticipatory grief in caregivers of elderly cancer patients. We verified the mediating role of family functioning and resilience in the relationship between caregiver burden and anticipatory grief. The findings support the proposed hypotheses as follows: (1) Caregiver burden has a direct positive effect on anticipatory grief. (2) Family functioning not only has a direct negative effect on anticipatory grief, but also plays a mediating role between caregiver burden and anticipatory grief. (3) Resilience has a direct negative effect on anticipatory grief, and plays a mediating role between caregiver burden and anticipatory grief. (4) Resilience moderates the mediating effect of family functioning.

The findings unveiled that family functioning and resilience partially mediate the relationship between caregiver burden and anticipatory grief. It revealed that caregivers with high levels of family functioning and resilience might perceive lower anticipatory grief despite the difficult caring circumstances resulting from heavy caregiver burden. Cancer treatment brings tremendous economic pressure, which may contribute to poverty or poverty reinstatement (Liu et al., 2022). In addition, caregivers may experience increased physical symptoms and declined body function due to caring work (Song et al., 2011). Well-family functioning provided by the family was significant to assist caregivers in reducing economic burden, maintaining social and mental health, and encouraging them to address issues actively, eventually improving their resilience (Hawkley et al., 2021), and leading to an adjustment in anticipatory grief (Coelho et al., 2018).

Caregivers of elderly cancer patients in this study showed a high level of anticipatory grief, which was higher than that reported by Li et al. (2022a). Their study was conducted on caregivers for adult Chinese patients with advanced lung cancer. On one hand, elderly cancer patients often have more severe physiological disorders, resulting in higher basic care needs and more prominent emotional problems. Thus, the caregivers of elderly cancer patients are under greater psychological stress (Sun et al., 2022). On the other hand, elderly cancer patients are more likely to have complications and closer to death than adult cancer patients. Caregivers of elderly cancer patients have a heightened sense of imminent loss of a loved one, which may result in a strong sense of loss and separation (Seyedfatemi et al., 2021).

Caregivers of elderly cancer patients in this study showed a moderate level of caregiver burden, which is higher than that reported by Menon et al. (2022). Their study was conducted in India, and most of the participants were caregivers for elderly patients with lung carcinoma and gastrointestinal cancers. The family-centered concept is deeply rooted in Chinese culture, and caregivers in China often regard caring for a family member as an obligation or responsibility, and thus are more likely to take on a lot of caregiving responsibilities. Since elderly cancer patients often have multiple comorbidities and complications, and are more likely to have more severe disease compared to adult cancer patients, it is more difficult for caregivers to cope with the care burden (Shin et al., 2018). Studies have shown that caregivers of elderly cancer patients are less likely to maintain a healthy lifestyle, such as regular exercise, healthy diet, adequate sleep, and actively seeking preventive health care, which increases their physiological burden (Lee et al., 2022).

In our study, both personal strain and role strain were found to be positive associations of anticipatory grief. There is a relationship between caregiver burden and anticipatory grief, with caregiver burden explaining about 36% of the variance in anticipatory grief, *F* = 6.343, *p* < 0.001, Adjusted *R*^2^ = 0.36. Elderly cancer patients tend to suffer from fatigue, loneliness, nausea, vomiting, diarrhea, weight loss, anxiety, pain, depression, and multiple morbidities (Kyriazidou et al., 2022). Therefore, caregivers need to provide long-term assistance with activities of daily living such as grooming, feeding, bathing, walking, and dressing (Bonin-Guillaume et al., 2022). Moreover, it is crucial to offer spiritual support and company to patients. Because of these demands, caregivers of elderly cancer patients are likely to experience a loss of personal freedom and inability to find adequate time for their personal needs (Li et al., 2022b), which can also cause anticipatory grief (Holm et al., 2019).

From a theoretical perspective, our study highlights the means to relieve caregiver burden in line with the conservation of resources theory. Based on the existing proposition, obtaining resources from environmental and internal factors can assist people in confronting stress or burden. Elderly cancer patients will require more support from caregivers as their health conditions change and their socioeconomic status deteriorates. Caregivers of elderly cancer patients are under a tremendous burden, such as physiological discomfort and emotional instability, and also need to incur heavy medical expenses (Jones et al., 2015). Therefore, provision of resources to caregivers can help them cope better with the caregiver burden. The social relationship is imperative for caregivers of elderly cancer patients. Chinese people tend to be self-confined when faced with problems, and they see family as an important source of social support (Yu et al., 2021). With the support of family members, caregivers of elderly cancer patients can maintain their social network and share the care responsibility, which may decrease their burden and improve emotional health (Steenfeldt et al., 2021). Caregivers of elderly cancer patients with high resilience are more likely to face caregiver burden with their toughness, and use a positive attitude to cope with the difficulties to reduce the impact of the caregiver burden (Bonanno, 2004). Therefore, when caregivers have higher family functioning and resilience, their caregiver burden and anticipatory grief would be alleviated.

In practice, nurses should develop policies and interventions to reduce caregiver burden and anticipatory grief. When an elderly cancer patient is diagnosed, nurses should provide support. Firstly, assessing caregivers who have the risk of burden, caring about their physiological, emotional, and social needs, and providing them personal psychological and financial support, and resilience skills (Baker et al., 2021). Secondly, nurses should counsel them that it is normal to feel sadness and grief, teach them how to deal with problems and manage stress, and help them access the available family support. Thirdly, nurses should guide caregivers to maintain health promotion-related behaviors, and maintain their physiological fitness to manage the caregiver burden. Finally, nurses should also participate in family activities, and encourage emotional communication among family members in these activities, so as to help them establish close relationships and improve family functioning (Kissane et al., 1998). In conclusion, nurses should pay full attention to the family functioning and resilience, and assist patients and caregivers in coping with cancer together, in order to promote caregivers’ role adaptation and relieve their anticipatory grief.

## Limitations and future directions

Some limitations of the present study should be acknowledged. Firstly, the study was conducted only in three hospitals in Western China, and only concerned the population of caregivers of elderly cancer patients, which may have caused a sampling bias and affected the representativeness of the findings. Secondly, whether participants had caregiving experience and different types of cancer of patients in sample selection was not adequately considered, which may also affect the level of clinical outcomes. Thirdly, the optimal cut-off values in this study were not confirmed. Finally, this study only focused on family functioning and resilience, while the demographic variables were not included in the structural equation modeling; therefore, additional variables should be considered in future research.

## Data availability statement

The raw data supporting the conclusions of this article will be made available by the authors, without undue reservation.

## Ethics statement

The studies involving human participants were reviewed and approved by the Ethics Committee of the School of Nursing, Lanzhou University in China. The patients/participants provided their written informed consent to participate in this study.

## Author contributions

CL: writing and original draft preparation. HZ and NT: review and editing. QZ and TY: supervision. HZ: project administration. LY, XP, and JW: collecting information. All authors have read and agreed to the published version of the manuscript.

## Funding

This study was supported by grants from the Natural Science Foundation of Gansu Province (No. 21JR7RA508), and the Fundamental Research Funds for the Central Universities (No. lzujbky-2021-34).

## Conflict of interest

The authors declare that the research was conducted in the absence of any commercial or financial relationships that could be construed as a potential conflict of interest.

## Publisher’s note

All claims expressed in this article are solely those of the authors and do not necessarily represent those of their affiliated organizations, or those of the publisher, the editors and the reviewers. Any product that may be evaluated in this article, or claim that may be made by its manufacturer, is not guaranteed or endorsed by the publisher.
